# *MySafeRx*: a mobile technology platform integrating motivational coaching, adherence monitoring, and electronic pill dispensing for enhancing buprenorphine/naloxone adherence during opioid use disorder treatment: a pilot study

**DOI:** 10.1186/s13722-018-0122-4

**Published:** 2018-09-24

**Authors:** Zev Schuman-Olivier, Jacob T. Borodovsky, Jackson Steinkamp, Qays Munir, Kyle Butler, Mary Ann Greene, Jonah Goldblatt, Hai Yi Xie, Lisa A. Marsch

**Affiliations:** 1 Center for Technology and Behavioral Health, Geisel School of Medicine at Dartmouth, Hanover, USA; 2 Department of Psychiatry, Harvard Medical School, Boston, USA; 3 Cambridge Health Alliance, Outpatient Addiction Services, Department of Psychiatry, Harvard Medical School, 26 Central Street, Somerville, MA 02143 USA; 4 Boston University School of Medicine, Boston, USA; 5 Tufts University School of Medicine, Boston, USA

## Abstract

**Background:**

While buprenorphine/naloxone (B/N) is approved for opioid use disorder treatment, effective delivery of B/N comes with significant challenges. Most notably, many patients do not take medication daily as prescribed; this non-adherence worsens treatment outcomes, increases healthcare costs, and leads to persistent worries of diversion among providers and policymakers. The present study examines the feasibility, usability, and acceptability of *MySafeRx*—a mobile technology platform integrating motivational coaching, adherence monitoring, and electronic pill dispensing designed to address the challenges of office-based opioid treatment (OBOT) with B/N.

**Methods:**

The *MySafeRx* platform integrates electronic pill dispensers, text-messaging, and videoconferencing to provide supervised self-administration of medication and daily motivational coaching through an Android app interface. High-risk early adults (18–39 years old) who were enrolled in OBOT with B/N and had documented illicit opioid use in the past month during opioid agonist therapy (n = 12) participated in a 28-day single-arm observational study of the *MySafeRx* platform in addition to standard care.

**Results:**

Two-thirds of participants who completed the study achieved an average of > 5 days per week of supervised B/N self-administration. Visual confirmation of medication adherence was demonstrated for an average of 72% of study days among all participants. All participants achieved platform technical proficiency within 60 min, reporting good levels of usability and acceptability. Illicit opioid abstinence rates confirmed by urine toxicology increased by 53% during *MySafeRx* but fell 43% within 3 weeks post-intervention.

**Conclusion:**

The *MySafeRx* medication adherence and remote coaching mobile platform is acceptable and can be feasibly implemented in real-world opioid use disorder treatment settings during high-risk periods (i.e., initial stabilization, after illicit opioid lapse), resulting in reduced illicit opioid use; however, the effect did not last after intervention completion, suggesting longer duration or extended taper of program may be needed.

*ClinicalTrials.Gov* NCT02942199 10/24/16 https://clinicaltrials.gov/ct2/show/NCT02942199

**Electronic supplementary material:**

The online version of this article (10.1186/s13722-018-0122-4) contains supplementary material, which is available to authorized users.

## Background

Opioid use disorder is a public health crisis. Opioid overdoses are the leading cause of death in the US for people under 50 [1], and more than 2.6 million Americans meet criteria for an opioid use disorder (OUD) [2]. Evidence-based opioid agonist treatments for OUD (i.e., buprenorphine, methadone) are currently utilized by healthcare practitioners to meet the growing treatment demand [3–5]. In particular, office-based buprenorphine treatment (OBOT) has been demonstrated to be an effective and safe means of treating OUD [6–8].

Despite the effectiveness of buprenorphine/naloxone (B/N) treatment [9–11], medication non-adherence still occurs. Masand, et al. define non-adherence as failing to take any prescribed doses (although patients who discontinue their medication after an initial period of adherence can also be correctly described as non-adherent), while full adherence is defined as taking all doses when and how they are prescribed. Partial adherence is defined as anything between full and non-adherence, including prolonged gaps in medication dosing, infrequent lapses, missed doses, incorrect dosing with less or more than prescribed, use of alternative route of administration, or change in dosing frequency [12]. Partial adherence and non-adherence are associated with reduced OBOT treatment retention [13], which in turn increases patients’ risk of relapse and overdose death [14]. In a Clinical Trials Network study, youth with OUD who ingested B/N on less than 5 out of 7 days per week (~ 71% of study days) had significantly lower odds of remaining in treatment at 12 weeks (OR: 0.07 (0.01, 0.89), *p* = 0.04). Similarly, OBOT patients who took B/N less than 80% of study days were 10 times more likely to relapse, and once patients left OUD treatment, the risk of opioid overdose increased rapidly [15, 16]. In addition, partial adherence to medication may be even more common than non-adherence with buprenorphine and leads to under-dosing [17]. Under-dosing not only increases rates of relapse and treatment dropout, but also increases opioid overdose susceptibility, as under-dosing medication may not achieve adequate mu-opioid receptor (MOR) blockade [18, 19]. Finally, adherence with B/N treatment reduces healthcare costs; one study showed a significant increase in total healthcare charges with < 80% adherence ($28,458 vs. $49,051; p < 0.01) [20], suggesting that medication adherence is critical for cost as well as outcomes.

The reasons for non-adherence can be multifactorial including patient-, provider-, and systems-level causes. On the patient level, motivational (e.g., ambivalence about recovery, treatment and medication), psychological (e.g. shame and stigma about medication [21], untreated psychiatric co-morbidity [22]), developmental (e.g. young age [23, 24], difficulty accepting chronic illness and the concept of maintenance treatment [24, 25], focus on identity, relationships, children, and work instead of treatment [26, 27]) and logistical (e.g., living with others who are using such as siblings [23], far distance to clinic [28], lacking money for co-pay) causes are common. Provider-level causes include discord between patients and providers or counselors leading to withdrawal, as well as insufficient clinical and administrative support to monitor and address non-adherence through measures such as observed induction, supervised administration of medication, pill counts, and toxicology testing. On the systems level, inadequate insurance reimbursements, frequent prior authorization requests and maximum dose limits, lack of easily accessible psychosocial treatment, burdensome co-pays, and a black market for diverted buprenorphine impact adherence [29, 30].

The problem of non-adherence is a common concern with many medications [31], but it is a particularly crucial issue in B/N treatment, because B/N is a Schedule III opioid medication and non-adherence often results in diversion, defined as “the unauthorized rerouting or misappropriation of prescription medication to someone other than for whom it was intended” [29]. Numerous studies suggest that prescribed B/N is often diverted to people without access to legitimate prescriptions who generally use it for self-treatment or withdrawal prevention [32–35]. Clinicians prescribing B/N believe that medication non-adherence is associated with increased diversion, by sharing or selling to friends and family [24]. Prescribers, who often have limited tools for preventing medication misuse and diversion, fear attracting DEA involvement [36–38], which could plausibly cause them to avoid prescribing patients higher doses of medication even when it may be needed. Although the Drug Addiction Treatment Act of 2000 (DATA 2000) enables waivered physicians to prescribe B/N outside of federally-licensed Opioid Treatment Programs, less stable and more complex OUD patients are commonly required to show up for daily monitored B/N dosing or referred to daily methadone maintenance. Similarly, current clinical practice guidelines recommend more frequent visits during the early stage of B/N treatment [39]. These treatment models often require patients to travel significant distances every day or several times a week to receive medication [28], which can interfere with patients’ abilities to maintain employment and provide childcare [2, 40] while they are already struggling with logistical and motivational barriers to participation in office-based treatment.

While mobile technology platforms have shown a moderate to high impact in enhancing chronic illness management and medication adherence in various primary care settings [41–44], limited experience with these platforms exists in OBOT settings. These multi-level gaps in treatment for unstable patients with OUD necessitate a low-cost intervention that can ensure daily B/N adherence, while also reducing medication diversion, enhancing treatment outcomes, and avoiding the increased time and travel burdens on patients caused by onsite supervised dosing programs [45, 46]. To provide a comprehensive solution to all of these issues, we developed the *MySafeRx* platform, a technology-based mobile intervention which integrates daily remote observation of medication self-administration, motivational recovery coaching, and secure electronic pill dispensers.

In this pilot study, we evaluated the feasibility, usability, and acceptability of the *MySafeRx* model for people with OUD in early adulthood (18–39 years old) who were prescribed B/N during periods of clinical instability. The primary aims of the present study were to demonstrate (1) feasibility of *MySafeRx,* by achieving supervised self-administration of B/N on > 5/7 days per week [13] for at least two-thirds of participants, with no reports of substantial B/N diversion; (2) usability, by having at least two-thirds of participants achieve at least 90% competency with the *MySafeRx* platform during 60 min of training and by having a mean usability of > 68, which is the cutoff of adequate usability on the well-validated system usability scale [47, 48]; and (3) acceptability, by achieving a mean overall participant satisfaction score of > 3 of 5 on a satisfaction scale at the end of the intervention period.

## Methods

### Study design

This was a single-arm, open-label clinical trial to receive 4 weeks of mobile recovery coaching and adherence monitoring via the *MySafeRx* program in conjunction with standard B/N OBOT. After signing informed consent, potential participants were asked to complete an initial survey battery to ensure eligibility. Eligible participants who were not excluded were invited to attend an in-office technology training session, which concluded with video-supervised self-administration of a B/N dose via the *MySafeRx* platform. We defined Day 1 of the study as the first day of video-supervised self-administration of a participant’s B/N dose via the *MySafeRx* platform. Participants who chose to start the intervention by partaking in the Day 1 technology training AND in-office, video-supervised self-administration of B/N (N = 12) were asked to complete weekly feasibility, usability, and acceptability surveys via REDCap [49] for 4 weeks. Weekly urine toxicology screens were obtained through the clinical treatment protocol during the 4-week intervention and then for 4 additional weeks to monitor the effects of *MySafeRx* discontinuation on illicit opioid use.

### Participants

Participants were recruited through referral from prescribers (n = 5) or OBOT nurse care managers (n = 2) in a regional OBOT network throughout the North Boston metropolitan region. Through regular email reminders and invitation of research coordinators to team meetings, OBOT providers were encouraged to refer unstable young adults who were struggling in OBOT while prescribed buprenorphine and at risk of termination from treatment OR who were recently inducted onto buprenorphine and felt to be at high risk of relapse in standard care. Those eligible were: (1) ages 18–39; (2) able to provide informed consent; (3) clinically diagnosed with opioid use disorder (DSM-5); (4) currently prescribed buprenorphine; (5) able to meet daily in a confidential place for scheduled videoconferencing; and (6) had an illicit positive opioid urine toxicology test in the past month or a missed urine toxicology with a self-report admission of past-month illicit opioid use while on opioid agonist treatment. Participants were excluded if they: (1) were unable to speak English or read the informed consent written at a 6th grade reading level; (2) were in their third trimester of pregnancy; (3) had cognitive deficits that may have limited their ability to complete study procedures; (4) had a medical requirement for twice-daily dosing of buprenorphine; (5) regularly used alcohol or benzodiazepines; or (6) exhibited signs of severe mental illness such as active suicidal ideation or psychosis. Participants were reimbursed with $60 for returning all the electronic devices, and up to $150 for study visit completion, including $30 for baseline visit, $30 for 2-week follow-up visit, $30 for 4-week follow-up visit, $2/day for completed check-in ($60 max).

### *MySafeRx* platform overview

The *MySafeRx* platform provides motivational support and medication adherence monitoring for people with OUD and mental illness during periods of vulnerability or instability (e.g., early B/N OBOT stabilization, after illicit opioid use lapse during B/N OBOT). Furthermore, it creates an integrated remote adherence monitoring and recovery support network by facilitating real-time confidential information sharing and communication between the site clinicians taking care of the participant (e.g., OBOT nurses, substance abuse counselors, and the prescriber) and the trained Mobile Recovery Coaches (MRCs) who check-in with participants each day.

The platform integrates four key components: (1) secure electronic pill organizers with unique medication release codes that are transmitted via Android app; (2) mobile text messaging, primarily including (a) programmed alerts which maintain and communicate contingency expectations from the underlying treatment program (e.g., “You missed your urine toxicology test this week. You are at risk of a medication hold in 48 h. Please complete the required testing and contact your doctor about next steps as soon as possible to prevent a medication hold.”) and (b) confidential communication with MRCs in order to initiate videoconferencing meetings (e.g., Are you available and in a private place for our video meeting within 5 min?”) or other communication consistent with motivational interviewing principles (e.g., emphasizing autonomy when someone doesn’t show up on video—“Taking your medication and what you want to talk about today is completely up to you, I will be available for the next 20 min if you want to meet.”); (3) daily remote videoconferencing check-ins, which include brief motivational interventions delivered by trained MRCs; and (4) a standardized protocol for supervised self-administration of medication via videoconferencing. These components, described in detail below, are integrated through the *MySafeRx* smartphone application and web portal. Initial proof-of-concept usability testing and iterative refinement of the platform took place for 1.5 years prior to this study with participants (n = 3) prescribed controlled substances for 3 months each during outpatient dual diagnosis treatment [50].

### Electronic medication dispenser and backup rescue lockbox

**Fig. 1:**
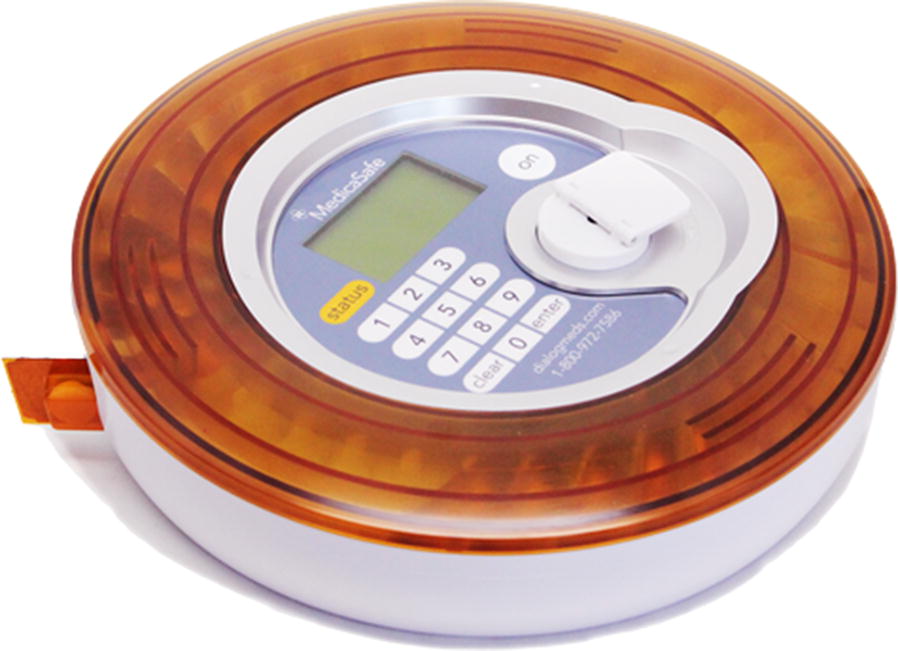
MedicaSafe pill dispenser

The MedicaSafe 3000 (www.medicasafe.com; Fig. 1) is a secure electronic medication dispensing device that uses unique random codes to unlock and dispense medication. MedicaSafe provided dispensers for the study free of charge. The *MySafeRx* system is designed so that the MRC can release this code to the participant during the adherence monitoring video encounter. The pill dispenser has the capacity to hold up to 30 days of medication (B/N tablets or films); however, for this study, the dispensers were refilled weekly onsite under the supervision of a registered nurse (RN) or medical doctor, and each MedicaSafe dispenser was filled with 6 days’ worth of medication. To address concerns about patient safety, a Masterlock 5900D combination lockbox, which costs $20, was provided to each participant with a 1-day rescue dose in case of technology failure, which gave sufficient time to resolve any issues arising during the study. During the pilot we had to change the safety protocol from 2 days to 1-day rescue dosing because of insurance provider constraints. The participant’s rescue lockbox code could be found by MRCs in the *MySafeRx* mobile application, allowing the MRC to release it in the case of dispenser failure or as the standard dose on the 7th day of each week. The rescue lockbox was refilled and the combination was reset and updated in the app at each weekly visit.

### Mobile recovery coaching, training, and certification

Mobile recovery coaches (MRCs) were responsible for conducting daily mobile medication adherence and recovery coaching sessions through the *MySafeRx* mobile application, using secure Wi-Fi or broadband connections from their residences, which were located in multiple US states. A remote *MySafeRx* manager was available 24/7 to support MRCs in the event of emergency, technology issues, or to provide backup when participants missed sessions and needed to dose outside the scheduled session window. These MRCs used the mobile application to coordinate with the local site clinicians directly responsible for the patient’s care.

Non-clinician mobile recovery coaches used a motivational interviewing (MI) framework for daily recovery coaching sessions. MI is an empathic, patient-centered conversational approach [51], which we selected as the theoretical background for the *MySafeRx* intervention because of its ability to focus on reducing ambivalence about taking medication and participating in treatment. All MRCs were required to pass a structured standardized patient interview designed by Dr. Theresa Moyers and Dr. Denise Ernst to assess MI competency using the Motivational Interviewing Treatment Integrity (MITI) Coding Manual 4.2 [52]. In order to be eligible for the 20-min standardized interview test, potential MRC candidates had to either already be Motivational Interviewing Network of Trainers (MINT) certified or had to participate in an introductory Motivational Interviewing (MI) training by *MySafeRx* staff and complete at least 6 weeks of supervised practice sessions with *MySafeRx* staff. After MRCs passed the MI competency interview test, participants were required to successfully complete a background check with references and a Criminal Offender Record Information (CORI) record check. Those who were successful then completed 2 h of interactive webinar training on how to use the *MySafeRx* platform and 90 min of safety and medication adherence training.

MRCs were scheduled to spend up to 840 min with each participant during the 4-week program. For this trial, we had 4 MRCs who worked on average 2–4 days per week. While the maximum daily capacity for a MRC was set at 10 participants, on the busiest day of the trial, the largest number of participants seen by a MRC during a single day was 4.

### Smartphone application and web portal

The *MySafeRx* Android application provided an encrypted (AES 128-bit) HIPAA-compliant vehicle for text messaging and daily adherence monitoring via video-conferencing sessions. All videoconferencing was conducted using the Zoom Application Programmer Interface and was transmitted using the encrypted Secure Socket Layer protocol with resistance to video freezing [53]. Unique meeting room IDs were generated and transmitted securely over HTTPS to the coach and participant’s Android devices.

The platform includes Android application user interfaces for both the MRC and the participant. The user interface for MRCs supports multiple functions: text messaging; session scheduling; initiating and conducting video sessions; recording medication adherence and substance use reports; releasing medication dose codes to open the dispenser; and securely communicating with clinicians and the *MySafeRx* manager. The participant user interface supports the following: text messaging; choosing a scheduled time to meet with a MRC; contacting the *MySafeRx* manager; and receipt of the dose code. The participant joins a video session with a MRC for motivational recovery coaching prior to receiving their medication each day, and the MRC uses the app to release the code needed to open the locked dispenser. Participants without an Android phone (n = 5) were provided for 30 days duration a 4G LTE LG Android G pad 7-inch tablet valued at $120, which was connected through a program “family plan” costing $160 per month to maintain up to 4 devices.

Prescribers and assigned site clinician delegates (e.g., team nurse or counselor) used the *MySafeRx* web interface to access details of their patients’ daily interactions with MRCs. The website provides clinicians with individual participant statistics, including medication adherence, drug/alcohol use, new physical or mental health symptoms, safety concerns, and triggers/high-risk events, reported daily by the MRCs in Daily Recovery Reports. We developed a software algorithm that produces a color-coded result for each day, representing various levels of concern about patient risk level: red (severe concern), yellow (moderate concern) and green (no concern). The color algorithm uses MRC-generated data to enable clinicians to rapidly digest information. Prescribers and delegates were responsible for responding in a clinically appropriate manner if notified of any non-adherence or clinically relevant symptoms. For non-urgent issues, prescribers can send messages through the website interface to MRCs as needed for addressing during the next morning’s dosing period.

### Daily workflow

**Fig. 2:**
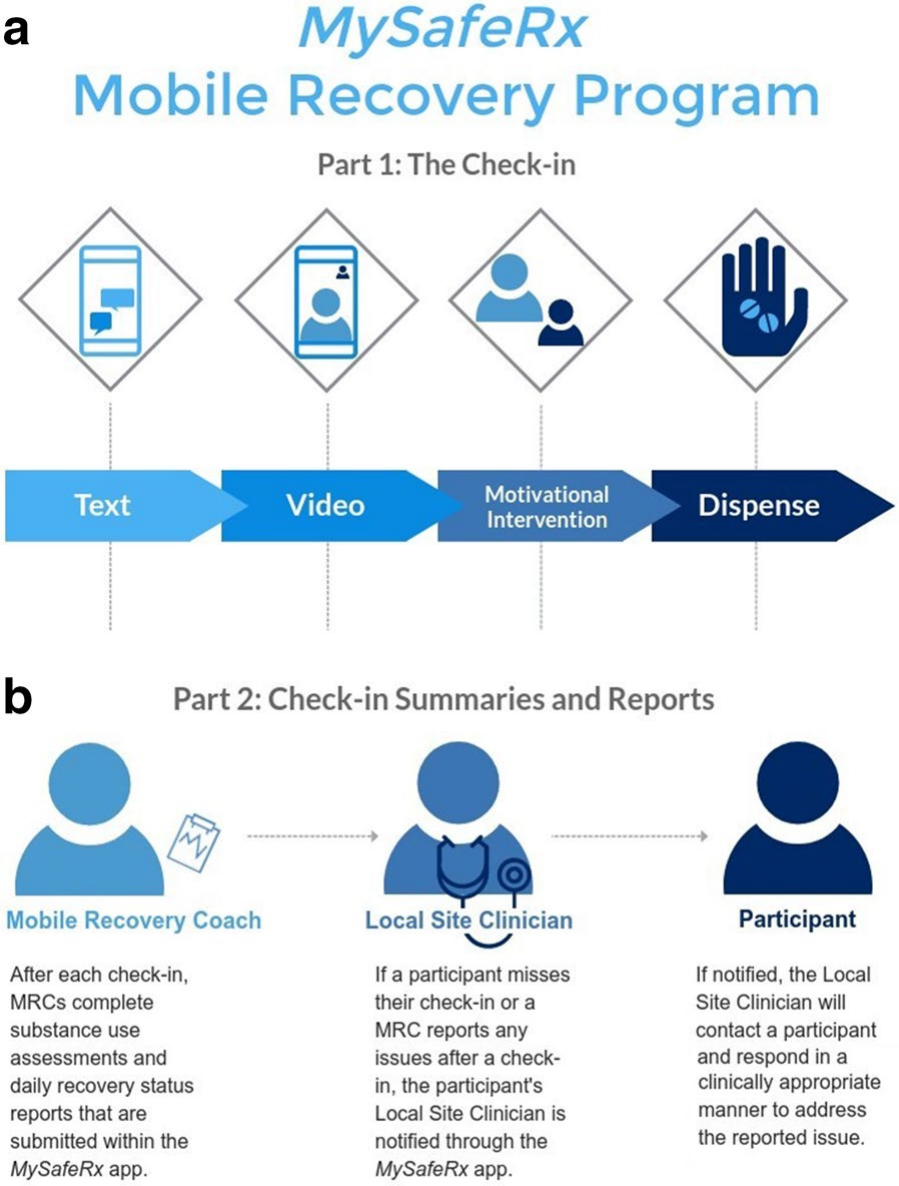
MysafeRx mobile recovery program

Each participant scheduled daily session times through the Android application with a MRC based on availability. In order to avoid the common tendency of patients to withdrawal from a program if conflict, discord, regret about something they did, or other negative transference emerges with a certain counselor [54], participants were encouraged to work with a minimum of three MRCs on different days throughout the trial, fostering a positive transference with the overall program instead of any one individual coach. Daily sessions began with a text reminder from a MRC sent through the *MySafeRx* application at the scheduled time, followed by a text invitation to begin videoconferencing (Fig. 2). Session duration ranged from 10 to 45 min and included a brief motivational intervention. During the session, the MRC sent an access code to the participant through the application, which allowed the participant to dispense their daily medication dose from a secure electronic pill dispenser (MedicaSafe 3000). The MRC observed medication placement and checked for dissolution in the mouth.

After the session, the MRC completed a Daily Recovery Report through the Android application, documenting the participant’s medication adherence, reported substance use, daily recovery goals, and other relevant notes. These reports were visible to other MRCs to allow collaborative team-based coaching and the clinicians who were clinically responsible for the participant’s treatment. Further, in the event of a serious safety concern, homicidal or suicidal ideation, the use of illicit opioids, or consecutive days of medication non-adherence, all clinicians on the patient’s care team were part of a rapid alert system that automatically notified them via email with a red-colored alert in the subject line. This email contained no protected health information (PHI); it merely advised all the participant’s clinicians to log into the secure web portal to view the details of the event.

### Safety and access to medication

In conjunction with the study Data Safety Monitoring Board, we developed management algorithms for MRCs and the *MySafeRx* manager to ensure that no patient who was motivated to take his/her medication was ever denied access to the medication. A backup MRC was always available if a participant missed their scheduled dosing time and the scheduled MRC was no longer available. We also opened an additional 4–5 PM scheduling window for participants who missed their morning scheduled time and who wanted to reschedule the dosing time. If someone was unable to join a videoconference (e.g., due to Internet connectivity issues), MRCs were instructed to provide the medication code by phone and to document this lapse in supervision of self-administration with a note to the participant’s prescribing clinician. The prescribing clinician would then make the clinical decision about the relevance of this as a non-adherence event. After each perceived non-adherence event, the *MySafeRx* manager would reach out to the participant by phone. When the next contact with the participant was established, the manager would confirm the nature of the missed daily recovery check-in. Finally, for participants who missed scheduled appointments, the MRCs were encouraged to focus future motivational enhancement sessions on program-interfering behaviors in order to help participants understand and overcome their barriers to attending appointments on time. The intention of this process was to help shape long-term attendance behaviors, which would benefit treatment both during and after the study.

### Measures

#### Baseline measures

Participants completed surveys about demographics, technology usage and attitudes [55], and substance use history. A doctoral-level provider conducted a psychiatric review of systems, using a DSM-IV SCID Screen [56], and the Montreal Cognitive Assessment [57] during the baseline screening visit. The Principal Investigator reviewed this information and the electronic health record for study participants, evaluating exclusion criteria, prior to scheduling the technology training and initial supervised dosing via the *MySafeRx* platform.

#### Primary outcomes measures

During the study, outcome measures were collected to assess acceptability, feasibility, and usability of the *MySafeRx* platform (NCT02942199).

1. Feasibility:
  - *Daily supervised adherence reporting* MRCs recorded through the application whether fully-supervised daily medication dosing and observed dissolution of medication in each participant’s mouth occurred.
    Our primary aim was to demonstrate *feasibility* by achieving supervised self-administration of B/N for an average of at least 5 of 7 days per week during the study for at least two-thirds of participants. This cutoff was based on a study of youth with OUD showing a marked difference in attrition for youth above and below 5 days per week of medication adherence [13].
  - *Self*-*report monitoring survey of non*-*adherence and diversion* We also assessed the level of self-reported non-adherence and collected reports about diversion reported by MRCs, providers, or participants. Participants completed a weekly survey via REDCap reporting whether they took all of their medication in the past week. If the answer was no, then they were asked to enter the number of days during the past week when medication was missed (non-adherence). Participants were also asked whether they shared or sold any medication in the past week. If the answer was yes, then they were asked to enter the number of days during the past week when medication was shared or sold to others (diversion). These self-report data were only visible to research staff, and not a participant’s referring prescriber and staff.
2. Usability:
  - *Technology training checklist (TTC)* After a maximum of 60 min of in-office training with the *MySafeRx* platform, study staff observed Day 1 in-office dosing session via the Android application and used the 10-item TTC to evaluate the participant’s competency with *MySafeRx* and the MedicaSafe Dispenser. We aimed to demonstrate *usability* by having at least two-thirds of participants achieve 90% competency during 60 min of platform training on the TTC by the second day of platform use.
  - *System usability scale (SUS)* [58] This reliable and valid measure of a system’s usability was completed by participants on Day 28. The scale is composed of 10 items on a 5-point Likert scale from “Strongly Disagree” to “Strongly Agree,” with half of the items being reverse coded [r]. Sample items include “I think that I would like to use this system frequently”; “I found the system unnecessarily complex [r]”; “I thought the system was easy to use”; “I found the system very cumbersome to use [r]”. The scale also included a section for open-ended comments. The scale is transformed following a standardized algorithm to provide a score ranging from 0 to 100, in which a 68 is the average score and is considered the cutoff for adequate usability (equivalent to a “C”) while 79 is the cutoff for excellent usability (equivalent to an “A−”), with 75–80 being the equivalent of a “B+” [47, 48, 59]. We aimed to demonstrate *usability* by having a mean score greater than 68 on the SUS after 28 days using the integrated platform.
3. Acceptability:
  - *MySafeRx satisfaction scale* After completing the study, participants were asked to fill out a 13-item system satisfaction scale with four principal components: ease of use, NOT bothersome, helpful, and NOT hindering treatment goals [60]. The survey used a 5-point Likert scale ranging from “Strongly Disagree” to “Strongly Agree.” We aimed to demonstrate *acceptability* with mean levels of overall satisfaction with the *MySafeRx* platform greater than 3 out of 5 after 28 days.
  - *Monitoring survey* This weekly questionnaire assessed overall acceptance of the integrated system by participant self-report. The survey used a 7-point Likert scale ranging from “Completely Disagree” to “Completely Agree.” We aimed to demonstrate *acceptability* with mean levels of overall satisfaction with the *MySafeRx* platform greater than 4 out of 7 after 28 days.
4. Impact on abstinence from illicit opioids by week:
  - *Urine toxicology screens* Nurse care managers conducted urine toxicology screening at least weekly for all participants using a clinical program protocol that included regular and random urine screening. Participants consented to have the results of all toxicology screens conducted by clinical staff during the study window be collected and stored in a database by study staff. Urine collection was conducted in a bathroom without a sink following standard clinic protocol but was not observed. Sample temperature was checked immediately, while specific gravity, pH and creatinine were analyzed for each participant to evaluate for evidence of tampering. Participants agreed to toxicology testing for buprenorphine, illicit opioids, amphetamine, benzodiazepines, alcohol, cannabis, and cocaine. Urine toxicology used enzyme-mediated immunoassay techniques (Beckman Synchron, Beckman Coulter, Fullerton, CA), but rapid chromatographic immunoassays were used for oxycodone (Bio-Rad, Hercules, CA) and Liquid Chromatography/Tandem Mass Spectrometry were used for fentanyl testing (LabCorp). Urine toxicology results were recorded by the laboratory in the EMR and these results were extracted into REDCap by study staff. The study team identified the most recent pre-study urine screen prior to Day 1, and then from Day 1 the analysis algorithm captured the next 8 weeks of required weekly clinical toxicology testing. Weeks with negative urine screens were considered as opioid-abstinent weeks for reporting outcomes.

### Data analysis

The primary outcomes (feasibility, acceptability, and usability) warranted primarily descriptive data analysis due to small sample size (n = 12), which was based on having a substantial cohort for usability testing determined by the 10 ± 2 rule [61]. We conducted secondary analyses on urine toxicology screening to understand the relationship between this medication adherence program and illicit opioid use. We evaluated whether the number of negative weeks of illicit opioid negative urine toxicology increased with time during the program, and if so, whether that increase would be maintained after completion of the 4-week *MySafeRx* program.

## Results

**Fig. 3:**
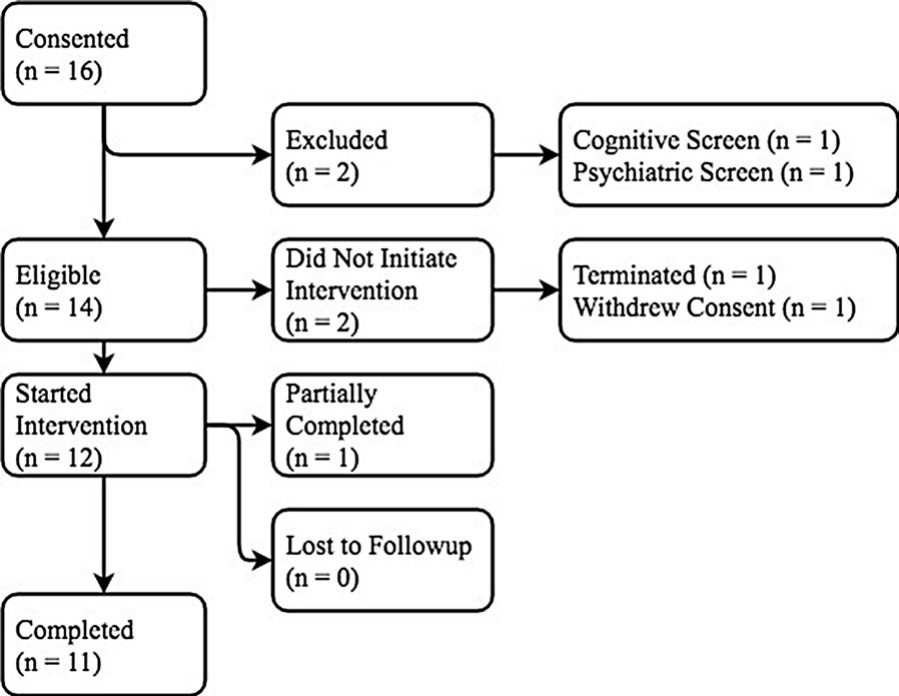
Consort diagram

Participants (N = 12) were primarily Caucasian and English-speaking with a mean age of 31.33 (range 26–36) (Fig. 3). Use of cocaine (33.3%) and benzodiazepines (41.7%) were common in the 30 days prior to study participation. Mental health comorbidity was also common: 58.3% of participants had an anxiety disorder, 41.7% had a major depressive episode in the past, and 25% had a diagnosis of PTSD (Table 1). Two participants had mental health comorbidity which prevented them from completing all baseline study activities. Prior to starting the intervention phase, one participant was terminated from the study and another withdrew consent.

**Table 1:** Sample characteristics

	Total	Male	Female
	N = 12	n = 8	n = 4
*Demographics*			
Age			
Mean (± SD)	31.33 (2.84)	31.13 (3.44)	31.75 (1.26)
[Range] years	[26–36]	[26–36]	[25–33]
Ethnicity, n (%)			
White	11 (91.7)	7 (87.5)	4 (100)
Other	1 (8.3)	1 (12.5)	0 (0)
Marital status, n (%)			
Single, never married	10 (83.3)	6 (75.0)	4 (100)
Married	2 (16.7)	2 (25.0)	0 (0)
Education, n (%)			
Some high school	3 (25.0)	3 (37.5)	0 (0)
High school graduate or GED	5 (41.7)	3 (37.5)	2 (50.0)
Some college or trade school	4 (33.3)	2 (25.0)	2 (50.0)
Employment, n (%)			
Employed	7 (58.3)	6 (75.0)	1 (25.0)
Unemployed	5 (41.7)	2 (25.0)	3 (75.0)
*Comorbid mental health disorder*			
Depression, n (%)			
Hx major depressive episode	5 (41.7)	3 (37.5)	2 (50.0)
Anxiety, n (%)			
Any anxiety disorder	7 (58.3)	4 (50.0)	3 (75.0)
PTSD, n (%)			
Subset of anxiety	3 (25.0)	2 (25.0)	1 (25.0)
*Recent illicit substance use*			
Cocaine, n (%)			
30 days prior to baseline	4 (33.3)	4 (50.0)	0 (0)
Benzodiazepines, n (%)			
30 days prior to baseline	7 (58.3)	3 (37.5)	4 (100)
*Technology use*			
Videoconference, n (%)			
Any use before study	4 (33.3)	4 (50.0)	0 (0)
Smartphone, n (%)			
Any use before study	12 (100)	8 (100)	4 (100)

### Feasibility

Two-thirds (8 of 12) of participants who completed the study achieved an average of > 5 days per week of supervised B/N self-administration. Mobile Recovery Coaches visually confirmed medication adherence for an average of 72% of study days for all participants. Ten of 12 participants reported perfect adherence via REDCAP self-report; however, dispenser data and coach reports demonstrated that only 1 of 12 had 100% adherence. No participant reported diverting medication by selling or sharing. Importantly, of the 12 study participants, only 10 returned their pill dispenser devices after 28 days (one reported it was stolen by her sister, while another was incarcerated, then moved out of town without returning it).

### Usability

All participants achieved 90% competency after the first training session, which lasted on average ~ 42 min (range 15–60). The average System Usability Score was 78.8 (SD 14.8, range 60–100) at the end of participation. Individual question means, and standard deviations are provided in Additional file 1: Table S1. Only three of twelve participants left feedback in the open comment question, two indicating that the app was “good” and “works very well,” and one indicating that the “app froze too often”.

### Acceptability

Overall satisfaction during the last week of the study as reported on the weekly Monitoring Survey report was on average 6.5 ± 0.7 (maximum 7). Participants endorsed being comfortable with using the pill dispenser (6.8 ± 0.4), the videoconferencing programs (6.7 ± 0.6), as well as the texting application (6.6 ± 0.6). Participants endorsed “seeing the benefit of taking my medication using the *MySafeRx* process” (6.5 ± 0.7), feeling the platform is “helping me become more independent” (6.5 ± 0.7), and has had a “positive influence on my recovery” (6.4 ± 0.8). Also, on the *MySafeRx* Satisfaction Scale delivered at the end of the program, participants displayed adequate overall satisfaction with a mean score of 4.3 ± 0.7 (maximum 5). At the end of the 4-week program, 9 of the 12 participants expressed a desire to continue with the *MySafeRx* program.

## Secondary analyses

### Toxicology

**Fig. 4:**
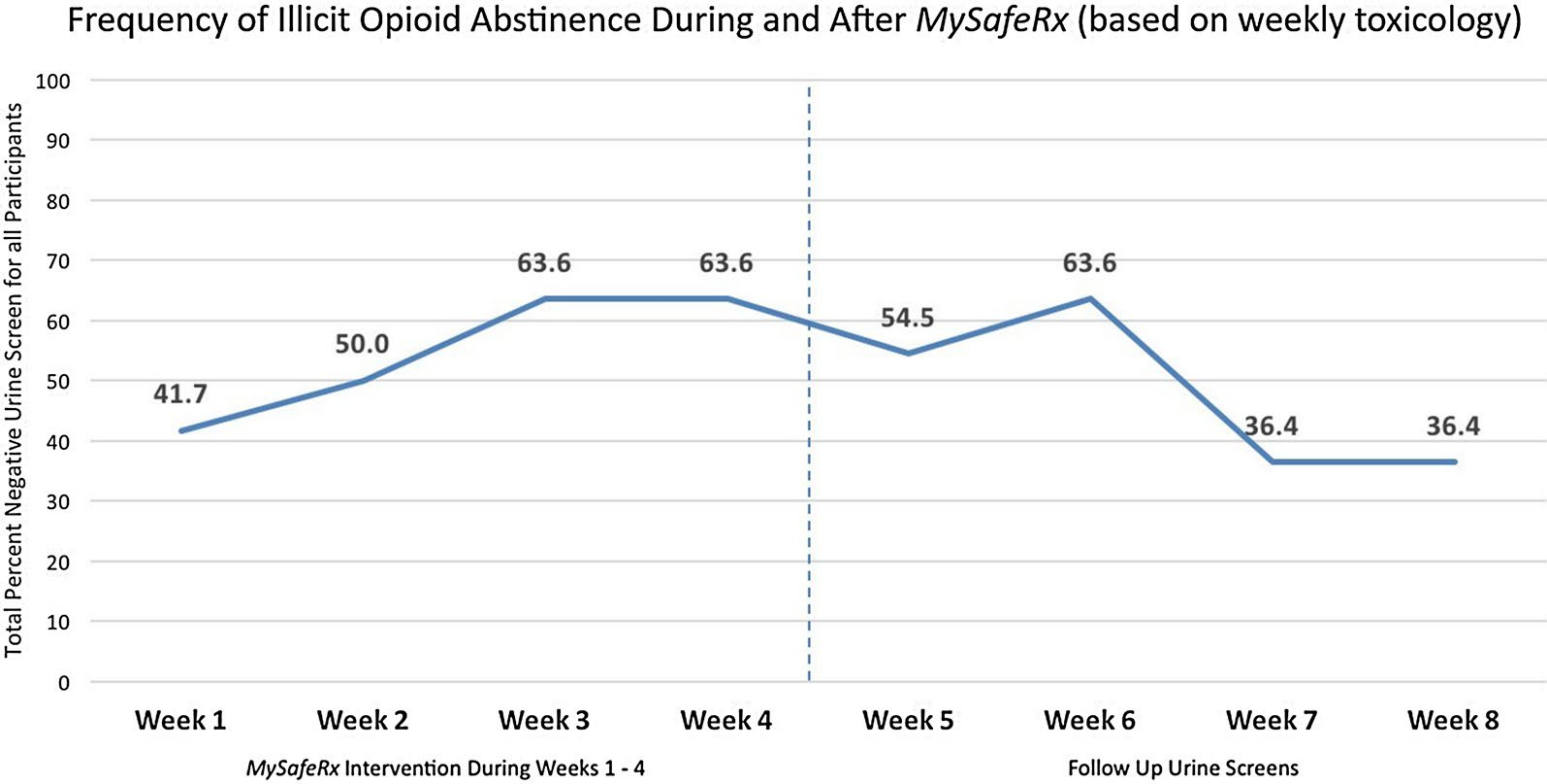
Frequency of illicit opioid abstinence during and after *MySafeRx* (based on weekly toxicology)

All participants (100%) had at least one urine toxicology screen positive for an illicit opioid other than buprenorphine in the 30 days prior to starting the study. The average level of illicit opioid abstinence, which was measured by percent negative toxicology (representing physiologic confirmation of no illicit opioid usage) per week started at 41.7% during week 1, then peaked at 63.6% for weeks 3 and 4 of the study during the intervention, representing a 53% increase in the frequency of toxicology-confirmed illicit opioid abstinence after 2 weeks of *MySafeRx*. However, within 2 weeks of discontinuing the *MySafeRx* program, the average percent negative toxicology fell to 36.4% by weeks 7 and 8, representing a 43% reduction in the frequency of toxicology-confirmed illicit opioid abstinence just 3 weeks after discontinuation of *MySafeRx* (Fig. 4).

### Adverse events

Two adverse events were reported during the study, and no serious adverse events were reported. The safety protocol indicated that emergency services should be contacted if suicidal or homicidal ideation is present, though this was not experienced in this pilot trial.

One participant’s family petitioned for involuntary commitment for substance use disorder treatment under MA General Law §35 [62] after the second week in the program while in the middle of a parental custody conflict. The participant was forced to taper off of buprenorphine, but completed post-study acceptability and usability surveys about the program. Another participant reported a domestic abuse situation during a *MySafeRx* daily coaching session. The participant’s study involvement was suspended while in crisis at a housing stabilization facility, which did not allow her to participate. She returned and completed participation in the study.

### Technology events

We found evidence that 19 missed scheduled check-ins out of 336 scheduled check-ins (5.6%) were a result of technology failure or patient’s loss of devices, and not related to ambivalence around medication taking. The *MySafeRx* manager was generally able to reach out to the patient outside of check-in hours by phone or meet the patient in the office. Two technology failures resulted in the participant only having a half dose of medication for the day: once because the lockbox was opened over the weekend; and once because after a phone crashed, the participant’s dispenser jammed getting second half of the day’s dose. The rescue box was used twice as a result of technology failure, both due to an error in the dose code generation for the MedicaSafe devices.

### Notable evening and weekend events with MySafeRx manager

In addition to the *MySafeRx* manager’s role with technology failure, device loss, and missed check-ins, the *MySafeRx* manager was accessible 24/7 by text and phone hotline, which played an important clinical role in several cases. There were three notable instances of remote *MySafeRx* manager contact with participants handling urgent issues that arose during evenings and weekends. The managers and MRCs were trained to respond to participant issues in accordance with severity: (1) One participant in emotional crisis sent a few erratic texts to the on-call manager on a weekend, who was able to alert the participant’s prescriber. The prescriber was able to reach out to the participant by phone, provide therapeutic relational support, and stabilize the situation by encouraging the patient to go to sleep. (2) In another instance a participant was heading to a public restroom in a restaurant where he had overdosed the week before with a new bag of fentanyl after having missed a planned meeting that weekend morning, when the *MySafeRx* manager reached out to the participant and talked with him until he flushed the drugs down the toilet, preventing a likely opioid overdose. The *MySafeRx* manager encouraged the participant to seek help with emergency services and paged the prescriber. (3) In the domestic violence situation mentioned above, the *MySafeRx* manager helped the participant find her way to the emergency department in the middle of winter with no belongings except her phone, where she was then able to get sent to a crisis stabilization unit for people with housing needs.

## Discussion

The *MySafeRx* platform provides remote medication adherence and motivational recovery support to individuals with OUD receiving OBOT with buprenorphine/naloxone. The results of the present study suggest that the *MySafeRx* platform can be successfully integrated and utilized within a real-world outpatient buprenorphine treatment setting to bolster treatment during periods of instability. These pilot data demonstrate that the *MySafeRx* platform provides consistent and reliable visual confirmation of patients’ buprenorphine self-administration. This study demonstrates that high-risk patients with OUD and co-morbid mental illness can learn to use the platform in a short amount of time, it is feasible to conduct training in a busy addiction clinic with overutilized space, and patients find the various components of the *MySafeRx* platform (e.g., app, pill dispenser) easy to use. Finally, the majority of participants were satisfied with this model of buprenorphine treatment delivery. In sum, *MySafeRx* is easy to use, amenable to patients prescribed buprenorphine in OBOT treatment and provides daily visual confirmation of B/N adherence without requiring substantial time or travel burden.

Should future studies confirm and extend these results to demonstrate robust clinical effectiveness, the *MySafeRx*-based buprenorphine treatment delivery model could potentially be utilized to address several opioid treatment-related shortcomings within the US healthcare system.

Taking the correct “blocking” dose of buprenorphine daily is important, because once buprenorphine binds to 90% of mu-opioid receptors (MOR) [63], it removes the possibility of euphoria or overdose from illicit opioids that day [19]—even most high-potency fentanyls [64]. For this reason, a daily patient-centered and motivation-focused intervention delivered during the critical moment of medication-taking each day could offer a new OBOT paradigm. Furthermore, taking the full dose of B/N as prescribed daily and reaching a steady-state can resolve dysregulation of the stress system that develops with chronic opioid use, and which contributes to ongoing depression, anxiety, and stress-related illness [65, 66] that can lead to relapse. By enhancing a person’s focus on recovery and treatment goals during the period of time surrounding B/N dosing, which has both the highest treatment receptivity and largest clinical impact each day, and providing daily structure and external accountability, an intervention would offer an untapped therapeutic opportunity and could increase medication adherence and treatment retention while reducing illicit opioid use, overdose risk, dysregulation of the stress system, and diversion [67, 68].

Second, only a limited number of physicians have received a waiver to prescribe buprenorphine, and many who have received a waiver do not fill their treatment capacity [69]. This hesitancy is likely the consequence of several factors including lack of necessary clinical resources to manage complicated patients, insufficient support networks for counseling and mental health, and fears about medication misuse and diversion [36–38, 69, 70]. Clinicians regularly take multiple time-consuming steps to reduce diversion of medication with abuse potential like buprenorphine, using staff resources and possibly reducing access to treatment [71]. Because the *MySafeRx* platform provides daily medication adherence monitoring and rapidly alerts prescribers to events that suggest clinical destabilization (e.g. high-risk drug use events, suicidal ideation, persistent absence from daily sessions, etc.), clinicians anecdotally reported to the study team that they felt more confident in their ability to manage high-risk OUD patients in their under-resourced setting. In an ongoing larger pilot trial among clinicians working in rural or underserved areas, where the availability of counseling support for primary care providers offering B/N has historically been more limited [72, 73], we are currently attempting to ascertain whether these anecdotal reports are supported with provider-level data. If providers feel this can help make treatment easier with their more difficult, high-risk patients, then *MySafeRx* might help contribute to a significant expansion of access to buprenorphine treatment by increasing the number of clinicians willing to prescribe buprenorphine and increasing access to buprenorphine in rural or underserved areas.

Third, non-adherence and partial adherence are associated with diversion behaviors, such as sharing and selling medication. One *MySafeRx* participant said, “I think the daily check-in and supervised taking of medication should be mandatory. Every person I’ve met on the Suboxone clinic sells their Suboxone and this will ensure they don’t.” Reducing opportunities for medication diversion can support medication adherence and improve treatment outcomes. Few existing office-based opioid treatment programs have the capacity for daily observed dosing needed to determine optimal stabilization doses for patients and prevent buprenorphine diversion. Other adherence technologies such as secure pill dispensers have recently been developed to address similar issues [74, 75]. However, without the ability to visually confirm medication adherence and dissolution with mouth checks, there remains a significant risk of diversion. Thus, stand-alone electronic pill dispensers may be an ineffective solution to address the rising rates of buprenorphine diversion. The daily medication monitoring provided by *MySafeRx* offers clinicians assurance that their patients are adherent to their medication and has the potential to support improvements in the patient-prescriber relationship and clinicians’ capacity to effectively and confidently gauge the impacts of dose adjustments.

Finally, efforts to disseminate OBOT in the US are complicated by the prevalence of non-evidence-based buprenorphine dosing schedules and fears about medication diversion. A recent meta-analysis suggests that higher doses of buprenorphine may result in better clinical outcomes [76]. Allowing patients to take lower doses multiple times per day (i.e., “split-dosing”), can reduce the duration of MOR blockade and thus complicates treatment [77]. Patients using this dosing schedule may be unknowingly “reserving the option” to achieve euphoria through use of high-potency illicit opioids later in the day. Additionally, while sub-therapeutic dosing should be avoided, certain concerned prescribers in areas of high diversion, who are wary about providing large drug supplies to unstable patients, may unintentionally prescribe patients sub-therapeutic doses. Such doses do not provide full MOR blockade [29] and may not be sufficient to provide the full blocking effect needed for increasingly potent and prevalent fentanyls [78]. This can limit the potential of the medication, challenge the therapeutic alliance, and lead to distrust on both sides of the doctor-patient relationship. For this reason, an intervention which includes daily supervised medication administration may help prescribers feel comfortable providing patients with higher, once-daily doses of medication needed to prevent relapse and overdose.

Importantly, there was a large discrepancy between objective adherence data (where just 1 out of 12 participants achieved 100% visually-confirmed adherence) and the self-report surveys (where 10 out of 12 participants reports full adherence every day). This points to the difficulty inherent in relying on self-report of medication adherence behaviors and emphasizes the importance of more robust forms of adherence monitoring.

In this study, abstinence from illicit opioids fell by 43% within 3 weeks post-discontinuation of the 28-day *MySafeRx* intervention, which was often accompanied by relapse and treatment drop-out. This increase in illicit opioid use after abrupt completion of the program is concerning and could be interpreted in several ways. First, the rapid decrease in illicit opioid use during the intervention suggests that the program enhanced adherence and reduced substance use, but that a longstanding change in behavior was not created with just 1 month of the intervention. Second, it is possible that a longer version of *MySafeRx* with a less abrupt transition back to standard care (an intervention ‘taper-down phase’) may reduce the risk of return to opioid use. Alternatively, like long-term maintenance in methadone maintenance treatment programs, it is possible that the program will only work when some or all the components are integrated and provided in a continuous manner. Further studies with longer time periods and various tapering schedules will be necessary to ascertain whether an episodic model or continuous maintenance model of remote daily supervised self-administration of B/N will be most effective for patients.

### Limitations

There were several limitations to this study. This pilot study had a small sample-size (n = 12) and was not designed to assess efficacy. Additionally, treatment dropout led to substantial amount of missing urine toxicology data. Finally, the self-report survey on medication adherence had two-step logic with an initial dichotomous question, which may have enhanced the probability of a social desirability bias resulting in over-reporting of medication taking and under-reporting of diversion by participants. The study found substantially lower adherence rates measured by supervised self-administration than by self-report on weekly monitoring surveys, which was expected. A final limitation is that this study did not assess prescriber and delegate attitudes about the intervention.

### Future research

Future studies with larger sample sizes are needed to evaluate efficacy outcomes, such as treatment retention, medication adherence, and illicit opioid use. In future studies, adding mobile technology to provide remote motivational engagement prior to medication treatment and to conduct observed induction are potential areas for future development. Additional data are needed to assess the impact of *MySafeRx* on both clinician attitudes toward increasing treatment capacity and medication doses as well as patient attitudes towards motivational mobile recovery coaching during daily dosing. In addition, cost analyses with larger samples will be important for evaluating actual real-world costs, which will be necessary in order to consider the potential for wide-scale dissemination. Patient, Mobile Recovery Coach, and clinician feedback should be incorporated into the design of future versions of the platform to further improve usability. Though roughly 63–76% of smartphone owners use an Android operating system [79] and Android had nearly an 82% market share worldwide in late 2016 [80], creating an iOS app version could provide even wider accessibility. Enhancing coordination with pharmacies and insurance providers as well as expanding platform accessibility are important challenges to address in the future to support dissemination.

Should future effectiveness trials yield positive results, the *MySafeRx* platform has the potential to provide the foundation for a nationwide adjunctive adherence support system for B/N treatment providers caring for vulnerable patients with OUD. In that case, it would be useful to evaluate whether the platform increases access to B/N treatment among high-risk groups. In conclusion, this study demonstrated the feasibility of integrating the MedicaSafe dispenser and *MySafeRx* and showed that the *MySafeRx* platform is acceptable to unstable young adults with OUD receiving buprenorphine treatment in a public-sector substance use disorder clinic.

## Additional file

**Additional file 1: Table S1.** System usability scale individual items (Mean ± Standard Deviation).
