# Supplementary material for: MySafeRx: a mobile technology platform integrating motivational coaching, adherence monitoring, and electronic pill dispensing for enhancing buprenorphine/naloxone adherence during opioid use disorder treatment: a pilot study
Source: Addict Sci Clin Pract. 2018 Sep 24;13:21. doi: 10.1186/s13722-018-0122-4 (PMC6154900; doi:10.1186/s13722-018-0122-4)
Supplement: Supplementary file 1 — Additional file 1: Table S1. System usability scale individual items (Mean ± Standard Deviation). [file 13722_2018_122_MOESM1_ESM.docx]

Supplemental Table 1

| **Question** | **Rating* (Mean ± SD)** |
| --- | --- |
| ­­­­­1. I think that I would use this system frequently. | - 1. ± 1.1 |
| 2. I found this system unnecessarily complex [r]. | 4.2 ± 0.4 |
| 3. I thought this system was easy to use. | 4.5 ± 0.6 |
| 4. I think that I would need assistance to be able to use this system [r]. | 4.3 ± 0.8 |
| 5. I found the various functions in this system were well-integrated. | 3.7 ± 1.1 |
| 6. I thought there was too much inconsistency in this system [r]. | 3.9 ± 1.1 |
| 7. I would imagine that most people would learn to use this system very quickly. | 4.3 ± 0.8 |
| 8. I found this system very cumbersome/awkward to use [r]. | 4.2 ± 0.9 |
| 9. I felt very confident using this system. | 4.4 ± 0.8 |
| 10. I needed to learn a lot of things before I could get going with this system [r]. | 4.2 ± 1.1 |

*All Ratings are adjusted to a 1 – 5 of 5 scoring with even questions reverse-scored [r] and inverted
